# Molecular Imaging and the PD-L1 Pathway: From Bench to Clinic

**DOI:** 10.3389/fonc.2021.698425

**Published:** 2021-08-23

**Authors:** David Leung, Samuel Bonacorsi, Ralph Adam Smith, Wolfgang Weber, Wendy Hayes

**Affiliations:** ^1^Translational Medicine, Bristol Myers Squibb, Princeton, NJ, United States; ^2^Technische Klinikum rechts der Isar, Technical University of Munich, Munich, Germany

**Keywords:** molecular imaging, PD-L1, PET tracer, radioisotope, ^18^F-fluorodeoxyglucose

## Abstract

Programmed death-1 (PD-1) and programmed death ligand 1 (PD-L1) inhibitors target the important molecular interplay between PD-1 and PD-L1, a key pathway contributing to immune evasion in the tumor microenvironment (TME). Long-term clinical benefit has been observed in patients receiving PD-(L)1 inhibitors, alone and in combination with other treatments, across multiple tumor types. PD-L1 expression has been associated with response to immune checkpoint inhibitors, and treatment strategies are often guided by immunohistochemistry-based diagnostic tests assessing expression of PD-L1. However, challenges related to the implementation, interpretation, and clinical utility of PD-L1 diagnostic tests have led to an increasing number of preclinical and clinical studies exploring interrogation of the TME by real-time imaging of PD-(L)1 expression by positron emission tomography (PET). PET imaging utilizes radiolabeled molecules to non-invasively assess PD-(L)1 expression spatially and temporally. Several PD-(L)1 PET tracers have been tested in preclinical and clinical studies, with clinical trials in progress to assess their use in a number of cancer types. This review will showcase the development of PD-(L)1 PET tracers from preclinical studies through to clinical use, and will explore the opportunities in drug development and possible future clinical implementation.

## Introduction

Programmed death-1 (PD-1) and programmed death ligand 1 (PD-L1) checkpoint inhibition plays a critical part in improving prognoses for patients with a range of tumor types (1). The immunosuppressive PD-1 receptor, expressed on various immune cells, including activated T cells, regulatory T cells, monocytes, and dendritic cells, is the target of a number of immune checkpoint inhibitors (ICIs), such as nivolumab and pembrolizumab (2). These treatments, together with those targeting the ligand PD-L1, which is expressed on both immune cells and tumor cells (3), increasingly form the backbone of immunotherapy for a variety of tumor types and disease stages (4). Optimization of these therapies relies on targeting patients who will most likely benefit from treatment (5). To achieve this goal, a better understanding of the underlying tumor biology is needed. Interrogation of the tumor microenvironment (TME) reveals considerable interplay between PD-1 and PD-L1 signaling (6), and although multiple molecules contribute to this immunosuppressive milieu, PD-L1 expression in some tumors is the single biomarker most closely associated with response to PD-1 blockade (6–8). Recent data indicate that low expression of PD-1 may also be associated with response to PD-1 blockade (9), although this requires further investigation.

PD-L1 expression may be predictive of benefit with ICIs (5). Currently, PD-L1 expression is assessed by immunohistochemistry (IHC) from tissue samples and is reported as a numerical value (percent positive tumor or immune cells). Therefore, a given result can only represent PD-L1 expression of a small portion of a selected tumor, and there are frequently multiple tumors (e.g., primary and metastatic sites) in the same patient that are not assessed (10). A way to start addressing this shortcoming is molecular imaging. Molecular imaging most commonly utilizes positron or single-photon emitting radionuclides to label specific targets, such as PD-(L)1 binding molecules, for *in vivo* visualization purposes (11–19). Using these positron emission tomography (PET) and single-photon emission computed tomography (SPECT) imaging techniques, PD-(L)1 expression of not just one part of a tumor, but of the entire tumor burden, can be assessed non-invasively (20, 21). Furthermore, molecular imaging allows serial monitoring of PD-(L)1 expression over time (20, 21), whereas temporal assessment by IHC is much more challenging clinically due to the requirement for multiple invasive biopsies (20, 21). In addition to safety considerations, other known limitations of IHC that could impact treatment decisions may include interobserver and intraobserver reproducibility, variability due to fresh *vs.* archival biopsied tissue (10, 22–24), and heterogeneity of expression within and among tumors (25, 26).

Molecular imaging therefore holds promise for *in vivo* quantification of PD-(L)1 expression in tumors and healthy tissue, as well as assessment of drug pharmacokinetics and pharmacodynamics (27), which will provide insights into the mechanisms of ICIs and ultimately improve patient selection, monitoring, and treatment (20, 21, 28). This review discusses the evolution of PD-(L)1 imaging from preclinical studies to current and potential future use in drug development and clinical settings, highlighting the opportunities for PD-(L)1 molecular imaging to improve healthcare outcomes.

## The Rationale for Molecular Imaging of PD-(L)1: Enhancing and Complementing Current IHC Assessment of PD-L1 Expression

Regulatory approvals of PD-(L)1 inhibitors have been accompanied by several companion or complementary IHC diagnostic tests to assess PD-L1 expression (5, 10, 29). However, methodological variations in scoring algorithms, cell types assessed (tumor cells, immune cells, or both) and expression cutoffs, as well as interobserver variabilities, can hinder data interpretation and reliability (30–32). Furthermore, heterogeneity in tumor PD-L1 expression within a tumor and among tumors within the same patient adds biological variation (26, 33). For example, Munari et al. showed that four or more biopsies were required to accurately evaluate and classify PD-L1 expression using IHC in patients with non-small cell lung cancer (NSCLC) (26). The situation is further complicated by the choice of tissue sample, with poor concordance between results derived from biopsy sections and whole tissue samples (25). Given the limitations associated with IHC-based PD-L1 assessment, alternative techniques such as molecular imaging and artificial intelligence (AI)-based digital pathology are being developed (20, 21, 28, 34).

### Clinical Utility of Current PET Tracers

PET imaging with the glucose analogue ^18^F-fluorodeoxyglucose (^18^F-FDG) is a technically mature imaging technique, and is a standard of care that is routinely employed for the diagnosis and monitoring of patients with cancer (35–38). In the context of immunotherapy, high pretreatment ^18^F-FDG uptake was associated with decreased duration of response and overall survival to ICIs in patients with melanoma (39, 40). In addition, on-treatment decreases in ^18^F-FDG uptake have been used as an early surrogate for clinical benefit (41, 42). For example, lower total lesion glycolysis and metabolic tumor volume (MTV), as determined in patients with NSCLC using ^18^F-FDG PET, have been shown to be prognostic and predictive of response and longer overall survival following nivolumab or pembrolizumab monotherapy (43–46). A metabolic score derived from combined assessment of pretreatment MTV and the neutrophil-to-lymphocyte ratio may provide a more accurate prediction of outcome than either of these factors alone (47, 48). Furthermore, in patients with NSCLC, the maximal standard uptake value (SUV) of ^18^F-FDG has been reported to be positively associated with PD-L1 expression (49, 50), and high ^18^F-FDG MTV is associated with PD-L1 expression ≥75% (48). However, ^18^F-FDG PET imaging, like computed tomography (CT), can show pseudoprogression (an early increase in tumor volume and FDG uptake on imaging with a subsequent favorable response to ICI therapy) (51, 52). Pseudoprogression is not common (approximately 5% of patients, according to current estimates) (53) but can potentially result in stopping effective therapy in individual patients (52). ^18^F-FDG PET has also shown promise in predicting response and overall survival in malignant melanoma and relapsed or refractory Hodgkin lymphoma after treatment with nivolumab and ipilimumab, an anti–cytotoxic T lymphocyte antigen-4 therapy (40, 54–56). Together, these studies indicate that ^18^F-FDG may facilitate the identification of patients most likely to benefit from ICIs (57). A number of novel PET tracers, such as ^11^C-choline, have also been investigated clinically and are reviewed in detail elsewhere (35, 36, 58, 59).

Within oncology, PET tracers allow the monitoring of biomarkers, such as PD-(L)1, across the whole body and over a treatment course, without the need for multiple biopsies (21, 60). The ability for isotopes to be conjugated to therapeutic antibodies, such as ^89^Zr-trastuzumab or ^89^Zr-nivolumab, may help to assess their pharmacokinetics. Studies have shown that patients who were trastuzumab-naive cleared ^89^Zr-trastuzumab at a faster rate than patients previously treated with trastuzumab (61, 62). A similar finding might be expected in patients being retreated or rechallenged with ICIs (63), an emerging patient population in the immunotherapy arena. A better understanding of the uptake and distribution of drugs/molecules and their mechanisms of action using molecular imaging may lead to earlier identification of potentially effective therapies and a consequent reduction in drug development costs (64, 65).

### Clinical Interrogation of PD-(L)1 PET Tracers

**Figure 1:**
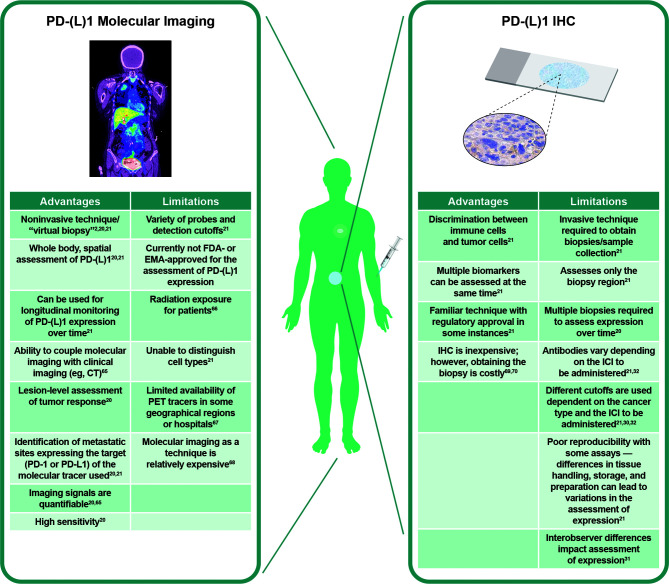
Visualization of PD-L1 expression at tumor sites. Left panel: PD-L1 PET tracers can be used to visualize and monitor PD-L1 expression at all tumor sites. Right panel: IHC can be used to assess PD-L1 expression at the biopsy site, representing a small region of only one tumor. Adapted from Du et al. (2) and Broos et al. (21). CT, computed tomography; EMA, European Medicines Agency; FDA, Food and Drug Administration; ICI, immune checkpoint inhibitor; IHC, immunohistochemistry; PD-1, programmed death-1; PD-L1, programmed death ligand 1; PET, positron emission tomography.

To understand the rationale of how specific PD-(L)1 molecular imaging may enhance and complement current IHC assessment of PD-L1 expression, it is important to consider in more depth the different advantages, limitations, and technical challenges of both techniques (**Figure 1**) (2, 20, 21, 30–32, 65–70).

It is likely that the most effective use of these techniques will be through a combination of the complementary information provided (71). Both IHC (30–32) and PET (71–73) are associated with issues regarding standardization of analysis and interpretation of results. PET imaging is less prone to preanalytical factors such as fresh *vs.* archival tissue samples, which may affect PD-L1 IHC assessment (74), or analytical factors such as different staining patterns between IHC assays (31). On the other hand, one tissue sample collected for IHC analysis can support evaluation of multiple biomarkers, cell morphology, and elements of the tumor and TME (21). Together, these technologies can augment each other: cellular and subcellular details from IHC complement the whole-body evaluation of tumor PD-(L)1 expression from molecular imaging (21). Imaging can thus serve as a “virtual biopsy” when tissue sampling is challenging; for example, during treatment or when the location of the tumor is unsafe for sampling.

Because cell types cannot be determined using PET, PD-L1 IHC and PD-L1 PET will likely become better aligned in IHC assays that assess PD-L1 expression on tumor and immune cells using the combined positive score algorithm (75, 76). With this in mind, we are optimistic that use of these techniques side by side will guide clinical decision-making in the future (76).

## Considerations for the Development and Use of PD-(L)1 PET Tracers

Considerations in developing molecular imaging agents such as PD-(L)1 PET tracers include the requirement for high target specificity and affinity as well as adequate tumor penetration of the tracer (77, 78). Furthermore, tracer uptake should have sufficient resolution to assess potential heterogeneity within each lesion and among lesions from the same patient (71–73).

Maute et al. (77) addressed affinity, specificity, and tumor penetration when determining whether PD-(L)1–directed immunotherapy could be improved with smaller, non-antibody therapeutics that could be radiolabeled and applied as a PET tracer. Binding affinity was investigated by identifying the key amino acid residues in the PD-1 ectodomain that are important to PD-1:PD-L1 interaction. The authors then engineered a high-affinity PD-1 variant (high-affinity consensus [HAC] PD-1) *via* selection of optimized mutation combinations, which led to a 15,000–40,000-fold increase in affinity and an increase in the half-life of the PD-1:PD-L1 interaction from ≤1 second to ~40 minutes (77).

A high degree of specificity of the radiolabeled ^64^Cu–DOTA–HAC–PD-1 for PD-L1 binding was confirmed by the lack of signal within PD-L1–negative tumors or in human PD-L1–positive tumors blocked by prior injection of unlabeled HAC–PD-1.

Due to its smaller size, tumor penetration was enhanced with ^64^Cu–DOTA–HAC–PD-1, showing binding to PD-L1 on tumor cells that appeared to be inaccessible to larger antibody binding (77). However, target binding affinity and specificity are only two factors influencing biodistribution of protein-based PET tracers *in vivo*. Other factors include protein size and glycosylation, metabolic stability, chelators, and the radiometal used for labeling (78, 79). The contribution of these factors on uptake by target and non-target tissues is complex and must be determined experimentally (78).

The choice of radionuclide deserves specific discussion. The half-life of the radionuclide on the molecular imaging agent needs to be compatible with the time needed for binding of the molecular target, while maintaining suitable levels of radioactivity to allow reasonable imaging resolution (80). Small biologics such as antibody fragments and adnectins show rapid distribution from vasculature to tissues (81, 82). An isotope with a short half-life (minutes to hours) is optimal (80), and imaging can typically occur during or soon after tracer administration. For example, ^11^C-acetate, originally employed in cardiology but now being used in oncology (in particular prostate cancer), has a physical half-life of approximately 20 minutes, and imaging is acquired shortly after tracer infusion (83, 84).

Clinical ^18^F-FDG PET (85) and ^18^F-BMS-986192 (72, 86) imaging of PD-L1 are both assessed at 60 minutes post-injection. Conversely, imaging with antibodies (e.g., ^89^Zr-labeled nivolumab) is best achieved 5 to 7 days post-tracer injection (72). However, imaging several days after tracer injection is inconvenient for the patient and makes it difficult to assess more rapid changes in the density of the target, as the tumor uptake on PET reflects the average density of the target during a period of 5 to 7 days. Furthermore, long-lived radioisotopes, such as ^89^Zr, cause a several-fold higher radiation exposure to normal organs than ^18^F (87). However, new total-body PET scanners will make it feasible to acquire PET scans with ^89^Zr-labeled radiotracers ~30 days after injection, and could allow for the radioactivity administered to be reduced by a factor of 40 (88–90).

In some instances, accumulation of tracers may be anticipated in a well-perfused physiological “antigen sink”, such as the spleen, which may result in insufficient uptake of the tracer in the tumor tissue (91). Co-administration of unlabeled versions of tracer may need to be investigated to reduce accumulation of the labeled tracer in antigen sinks (11). If the imaging agent is derived from a therapeutic agent (11), an analogous investigation may be warranted.

The dynamic range of a tracer is a further parameter to consider, as is the proportion of signal alteration that can occur under a perturbed system; for example, altered expression of a biomarker in a disease state. A high dynamic range allows smaller alterations to be accurately detected, thus increasing sensitivity (92). In general, PET is considered to be better suited than SPECT for tracer quantification and in dynamic imaging (65). SPECT is markedly less sensitive than PET and accurate quantification of activity concentrations is challenging (65). Due to lower sensitivity, SPECT scans take longer than PET scans (93); therefore, as a result of the additional burden on patients and resources, repeated SPECT imaging may be less well tolerated than repeated PET scans. The dynamic process of tracer uptake and retention can be better assessed through a time series of images during dynamic imaging, as opposed to a single time point from a static image (94).

In addition to the biological and chemical considerations around development of a PET tracer, it is important to describe the quantification procedure, including reporting SUVs or tumor:blood pool ratios, for example (71–73). However, the uptake of a radiotracer depends not only on binding to its target (specific uptake), but also on other mechanisms (non-specific uptake) (95). Therefore, quantifying target expression with a simple image-derived parameter such as an SUV or a tumor:blood pool ratio may not be optimal. Dynamic whole-body imaging, in contrast to static imaging, can often produce multiparametric images of influx rate and distribution volume while also providing conventional “SUV” equivalents (96). Systematic studies are needed to provide a quantitative parameter to allow for a best estimation of tracer concentration in the target tissue.

## From Preclinical to Clinical Studies Using PD-(L)1 PET Tracers

The *in vitro* and *in vivo* preclinical models used to test PD-(L)1 PET tracers have grown in complexity, from early studies using cell-line–based systems and mouse models (14, 16, 19, 77), including xenografts and other tumor models, to more recent studies using healthy non-human primates (11, 12) (**Supplementary Table 1**). Results from preclinical studies of PD-(L)1 PET tracers have been encouraging, with tracers demonstrating specific binding and the ability to detect varying levels of PD-(L)1 expression, including endogenous expression (14, 17–19, 77, 97, 98). Studies in more complex systems such as non-human primates have involved both ^18^F-BMS-986192, an ^18^F-fluorine labeled anti–PD-L1 adnectin small molecule, and ^89^Zr-nivolumab (11, 12). Further imaging studies in healthy cynomolgus monkeys using ^18^F-BMS-986192 and ^89^Zr-nivolumab, which specifically bind to PD-L1 and PD-1, respectively, have begun to directly investigate whether PD-(L)1 imaging could be a viable option for imaging in humans (11, 12). As noted, binding of ^89^Zr-nivolumab in the PD-1–rich spleen “antigen sink” could be reduced by co-administration of unlabeled nivolumab (11). ^18^F-BMS-986192 has low to moderate uptake in the lungs, heart, liver, and muscles. This biodistribution allows good contrast with PD-L1–positive tumors (12). ^18^F-BMS-986192 does have an expected higher tracer uptake in the spleen and in urinary structures (excretion pathway), although these are not common organs of metastatic disease in solid tumors. Radiation dosimetry indicated that this tracer was safe to administer in humans (12).

These preclinical studies suggest that PD-(L)1 PET imaging is a viable technique to assess PD-(L)1 expression in humans, and PD-(L)1 PET tracers have progressed into first-in-human studies (**Table 1**). These first-in-human studies have provided insights into various aspects of molecular imaging, including biodistribution, intratumoral and intertumoral heterogeneity, and preliminary safety findings, including those indicating a lack of toxicity (71–73, 100).

**Table 1:** Examples of clinically tested PD-(L)1 PET tracers.

Author, year	Tracer	Biodistribution/accumulation	Key findings
Niemeijer et al., 2018 (72)	^18^F-BMS-986192	Both tracers showed high accumulation in the spleen and liver. ^18^F-BMS-986192 showed some uptake in the hypophysis	No tracer-related adverse events of grade ≥3 No accumulation of ^18^F-BMS-986192 and ^89^Zr-nivolumab occurred in normal brain Heterogeneity in the uptake of ^18^F-BMS-986192 occurred between and within patients Accumulation of ^18^F-BMS-986192 and ^89^Zr-nivolumab was seen in some, but not all, brain metastases ^18^F-BMS-986192 uptake in tumor lesions correlated with PD-L1 expression assessed using IHC. The uptake of ^89^Zr-nivolumab correlated with PD-1–positive, tumor-infiltrating immune cells Response to nivolumab evaluation on a lesional basis (excluding lesions <20 mm diameter) showed that ^18^F-BMS-986192 SUV_peak_ was higher for responding lesions than non-responding lesions (median 6.5 *vs.* 3.2, *P* = 0.03, Mann–Whitney U-test) (analogous lesional correlation for ^89^Z-nivolumab median SUV_peak_ 6.4 *vs.* 3.9, *P* = 0.019, Mann–Whitney U-test)
	^89^Zr-nivolumab		
Bensch et al., 2018 (73)	^89^Zr-atezolizumab	High uptake over time occurred in the intestines, kidneys, and liver. Low uptake occurred in the brain, subcutaneous tissue, muscle, compact bone, and lungs	Lesions at all main metastatic sites were visualized The detection of CNS lesions was not determined, as patients with CNS metastases were excluded from the study Within-patient heterogeneity was observed in patients with >1 lesion Heterogeneous intratumor tracer uptake was observed One low-grade adverse event was reported ^89^Zr-atezolizumab uptake increased with SP142-assessed PD-L1 staining, but not with SP263-assessed PD-L1 expression
Xing et al., 2019 (71)	^99m^Tc-NM-01	Biodistribution was observed in the kidneys, liver, and spleen, and to a lesser extent in the bone marrow and lungs, reflecting the physiological expression of PD-L1	Intratumoral and intertumoral heterogeneity was observed Acceptable dosimetry was reported, with levels similar to other agents in clinical use No drug-related adverse events were reported Primary tumor:blood pool ratios at 2 h correlated with IHC
Verhoeff et al., 2020 (99)	^89^Zr-durvalumab	High ^89^Zr-durvalumab retention was observed in the spleen and liver	Heterogeneous accumulation was observed within tumors and between patients Uptake of ^89^Zr-durvalumab was not seen in all ^18^F-FDG–positive tumors No correlation between tumor PD-L1 expression determined using ^89^Zr-durvalumab uptake and PD-L1 expression on archival tissue was found
Huisman et al., 2020 (100)	^18^F-BMS-986192	–	In PD-L1–positive lesions, time–activity curves for ^18^F-BMS-986192 increased over time, while for PD-L1–negative tumors the time–activity curves remained approximately flat up to 40 minutes post-injection At 60 min post-injection, in 61% of the tumors analyzed, the uptake of ^18^F-BMS-986192 was best described by a reversible single-tissue model. In 39% of the tumors analyzed, including in one lesion with a 10% positive IHC score and in one lesion with a negative IHC score, an irreversible two-tissue model was preferred SUV normalized to injected activity over body weight correlated best with the distribution volume of ^18^F-BMS-986192

CNS, central nervous system; FDG, fluorodeoxyglucose; IHC, immunohistochemistry; PD-1, programmed death-1; PD-L1, programmed death ligand 1; PET, positron emission tomography; SUV, standard uptake value.

In terms of biodistribution, accumulation in the spleen and bone marrow is an expected feature of PD-(L)1–targeting agents (11, 16, 101). As mentioned, the accumulation of antibodies in “antigen sinks”, such as the spleen, may require the use of an unlabeled version to block antibody binding sites to allow more of the labeled tracer to reach the tumor (11, 16, 101). First-in-human PET with ^18^F-BMS-986192 and ^89^Zr-nivolumab reported tracer uptake in the spleen and marrow (72). Whole-body PD-(L)1 PET-CT with ^89^Zr-atezolizumab also accumulated in the spleen, with uptake also occurring in the bone marrow over time (73). In contrast, the accumulation of tracers is low in the lung and absent in healthy brain (11, 12, 72).

Although PD-(L)1 PET tracers have not been shown to accumulate in the brain (72, 73), a key outstanding question is whether PD-(L)1 PET can be used to image brain metastases that typically cause a disruption in the blood–brain barrier. Central nervous system uptake of ^18^F-BMS-986192 and ^89^Zr-nivolumab was observed in some, but not all, of the untreated brain metastases in two patients enrolled in the first-in-human study (72). Further studies addressing the ability of tracers to cross the blood–brain barrier will be required and should ideally include pathologic correlations.

Given the spatial and temporal nature of molecular imaging, an additional benefit for patient outcomes is the identification of multiple tumor sites. Results from first-in-human trials using ^89^Zr-atezolizumab, ^18^F-BMS-986192, ^89^Zr-nivolumab, and ^99m^Tc-NM-01 indicated that PET tracers can also be used for the visualization of multiple lesions expressing PD-(L)1 (71–73). Tracer uptake heterogeneity was found to be relatively common among metastases (71, 72).

Alongside biodistribution data in humans, safety data are also imperative. Toxicity and safety data from the first-in-human studies revealed no reported tracer-related adverse events for ^18^F-BMS-986192, ^89^Zr-nivolumab (72), or ^99m^Tc-NM-01 (71), although one grade 3 infusion-related adverse reaction was reported in the first-in-human study using ^89^Zr-atezolizumab (73).

Given the association between IHC-determined PD-L1 expression and response to ICIs, it is crucial to demonstrate that a similar relationship exists for molecular imaging. Although there are limited data available showing association of PD-L1 expression assessed by molecular imaging with efficacy, encouraging results were provided by the first-in-human study of ^89^Zr-atezolizumab, in which clinical response was better correlated with pretreatment PET-imaging–assessed PD-L1 expression compared with either IHC-based or RNA-sequencing–based PD-L1 assays (73). Similarly, Niemeijer et al. demonstrated that PD-(L)1 PET imaging can predict lesion-level response (**Table 1**) (72). Moreover, tumors determined to be PD-L1–positive by IHC and PET accumulate nivolumab, while PD-L1–negative tumors do not (72). These findings demonstrate that tumors that use PD-L1 for immune escape can be readily targeted by nivolumab immune blockade (72). This also suggests that PD-L1 determination by molecular imaging or other methods may facilitate the selection of patients who are most likely to respond to treatment with ICIs.

### Affirming the Use of PD-L1 PET Tracers in Clinical Studies: Assessment of PD-L1 Expression by IHC and Molecular Imaging

It is of interest to assess whether any new PD-L1 assessment using molecular imaging in preclinical and clinical studies shows consistent imaging and biodistribution of PD-L1 when compared with the current standard assessment by IHC, taking into consideration the nuances and variations already described with IHC analyses. Agreement between PD-L1 IHC and molecular imaging has been assessed in a number of preclinical studies and has generally been found to be high, although most studies do not report statistical concordance assessments (12, 13, 17–19). This includes results with the PD-L1 PET tracers ^111^In-MPDL3280A and NIR-MPDL3280A in triple-negative breast cancer (TNBC) and NSCLC xenografts (19), and with ^18^F-BMS-986192 in NSCLC (12). Consistent imaging and biodistribution was also reported between IHC-determined PD-L1 expression and the uptake of the PD-L1 PET tracer ^111^In-DTPA-anti-PD-L1 in xenografts and mouse models (16).

Correlation between PD-L1 imaging and IHC was reported in the first-in-human trials (71–73). Niemeijer et al, in a study assessing the entire PD-1 and PD-L1 pathway, reported correlation of the PET signal of ^18^F-BMS-986192 with PD-L1 IHC and of the ^89^Zr-nivolumab PET signal with PD-1 IHC. The median ^18^F-BMS-986192 SUV_peak_ was higher for lesions with ≥50% tumor PD-L1 expression by IHC than for lesions with <50% (8.2 *vs.* 2.9, *P* = 0.018, Mann–Whitney U-test) (72). In a study by Bensch et al. (73), uptake of ^89^Zr-atezolizumab was higher in lesions with IHC-determined PD-L1 expression than in those without. However, this was only the case with the Ventana PD-L1 (SP142) assay, not the Ventana PD-L1 (SP263) assay, highlighting that variations among IHC assays should be taken into consideration. This study also found that tracer uptake differed between tumor types, with TNBC showing an average 50% less uptake than locally advanced or metastatic bladder cancer (73). Tumor vascularity may be related to tracer uptake and could account for some uptake differences between tumor types (102). Concordance between primary tumor:blood pool ratios of a SPECT-based tracer and IHC has also been reported, with primary tumor:blood pool ratios at 2 hours correlating with PD-L1 IHC (*r* = 0.68, *P* = 0.014) (71). Dosimetry for ^99m^Tc-NM-01 was reported to be similar to other SPECT agents in clinical use (71), indicating that the use of ^99m^Tc-NM-01 in patients is feasible. Furthermore, quantitative assessment with this tracer in patients with NSCLC has been demonstrated to be reproducible and reliable between independent observers (103). The results from these studies indicate that PD-(L)1 molecular imaging generally shows concordance with IHC-based PD-L1 expression. However, as concordance was not observed in all studies, some caution is required.

### The Growing Momentum for PD-(L)1 Molecular Imaging Clinical Studies

**Figure 2:**
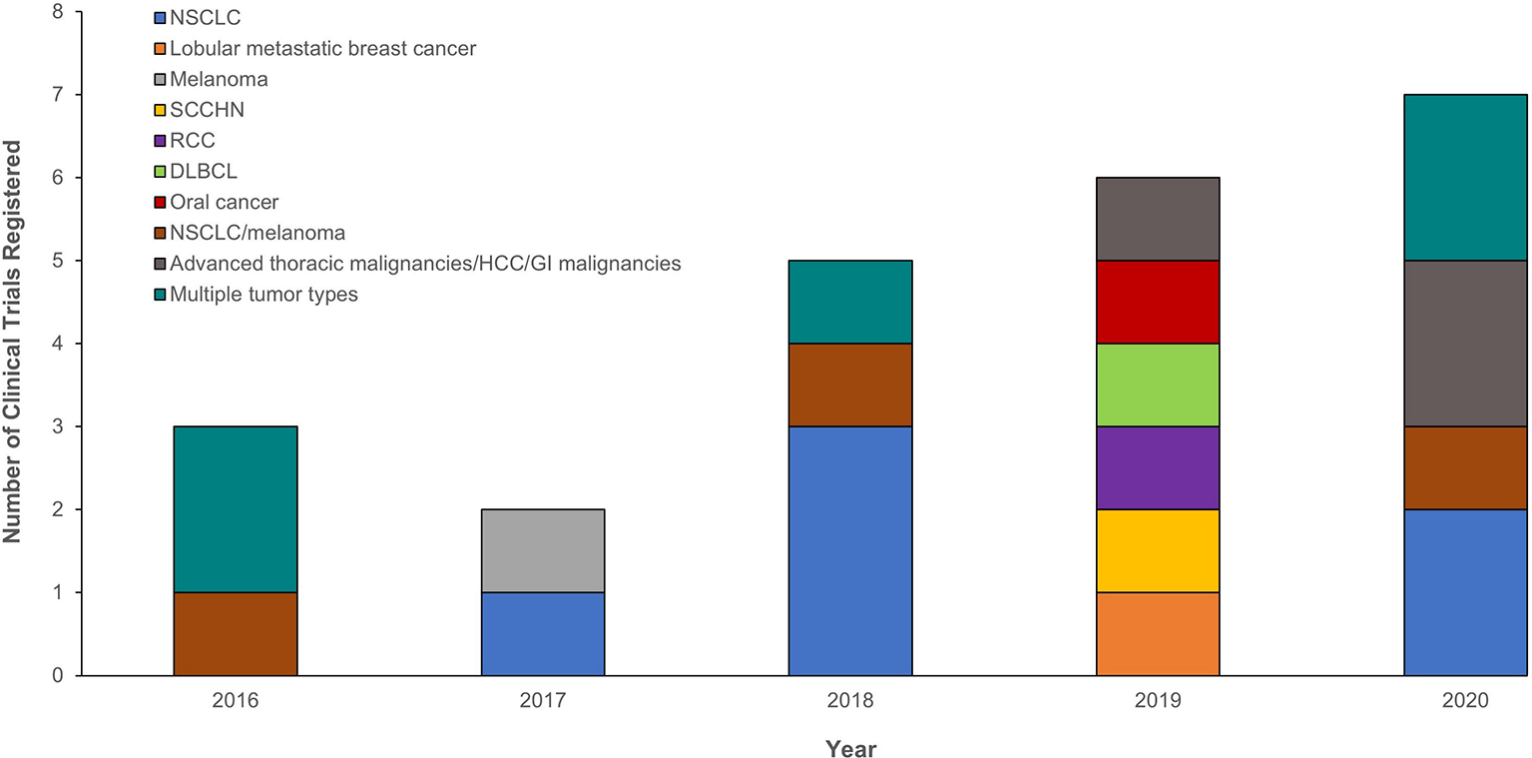
Registered clinical trials between 2016 and 2020 evaluating the potential role of PD-(L)1 PET tracers in assessing PD-(L)1 expression and the tumor types investigated. Only trials registered with ClinicalTrials.gov are presented at the year the trial was initiated. The following search term was used for each year: (“nuclear medicine” OR imaging OR 89Zr OR 18F OR 99mtc) AND (PD-L1 OR PD-1 OR anti-PD-L1 OR anti-PD-1). DLBCL, diffuse large B-cell lymphoma; GI, gastrointestinal; HCC, hepatocellular carcinoma; NSCLC, non-small cell lung cancer; PD-1, programmed death-1; PD-L1, programmed death ligand 1; PET, positron emission tomography; RCC, renal cell carcinoma; SCCHN, squamous cell carcinoma of the head and neck.

Several clinical trials evaluating the potential roles of PD-(L)1 PET tracers in assessing PD-(L)1 expression are recruiting or active. Although trials were initially undertaken in NSCLC, the progression of ICI use into other tumor types has seen the expansion of PD-(L)1 PET tracer studies into squamous cell carcinoma of the head and neck, breast cancer, renal cell carcinoma, diffuse large B-cell lymphoma, melanoma, and other cancers (**Figure 2** and **Supplementary Table 2**). These trials aim to validate the initial proof-of-principle, first-in-human studies with larger datasets. The majority are being performed in single or multiple institutions with industry sponsorship; however, for rare tumors, collaborative groups across multiple institutions may be advantageous. Depending on the critical clinical question being addressed, the trial design may vary. Studies addressing pharmacodynamic changes of a biomarker will need to have multiple scanning time points (e.g., NCT03850028; **Supplementary Table 2**), whereas those that aim for baseline biodistribution may have a single scan (e.g., NCT03564197, NCT02978196; **Supplementary Table 2**).

The further development of PD-(L)1 molecular imaging as a clinical research tool and biomarker requires several important steps. The first is a body of evidence supporting the clinical utility of PD-L1 PET as a diagnostic tool and comparing it with standard methods for evaluating PD-L1, such as IHC. Several prospective trials are ongoing to determine a predictive or prognostic benefit of molecular imaging for ICI therapy (e.g., NCT03514719, NCT03564197, and NCT03843515; **Supplementary Table 2**). Second, there is a need to adopt standardized imaging protocols and criteria for quantitative image analysis (57). Involvement of industry collaborations or large institutions may be necessary to acquire the necessary data and achieve harmonization. Third, as noted above, imaging with radiolabeled antibodies results in effective radiation doses that are several-fold higher than for PET imaging agents, such as ^18^F-FDG and ^68^Ga-DOTA-TATE (104). These radiation doses may limit broader use of PD-(L)1 imaging, especially for serial PET scans to monitor changes in PD-(L)1 expression in individual patients. Development of PET imaging agents labeled with short-lived positron emitters, such as ^18^F or ^68^Ga, is a highly active research field with encouraging preclinical and clinical results (12, 72, 105). PET imaging studies generally use micro-dosing, defined as less than one hundredth of the dose that has a pharmacological effect, to a maximum of 100 µg, which is considered to have a very limited risk to participants (106). This strategy is used in NCT02978196, for example (**Supplementary Table 2**). Further trials should follow guidelines, such as those provided by the United States Food and Drug Administration for radiolabeled PET tracers (106), to be approved for clinical implementation. It is anticipated that ongoing and future trials will provide the solid body of evidence necessary to develop guidelines for the adoption of molecular imaging into routine clinical practice.

## Discussion

There are opportunities and challenges facing the incorporation of molecular imaging for PD-(L)1 expression into drug development and routine clinical practice. Some of the key scientific questions relating to safety, correlation with IHC, and prediction of patient outcome are being investigated, as summarized above. Data from studies investigating the impact of PD-(L)1 imaging on patient outcomes are not yet available. For broader clinical use of PD-(L)1 imaging, it will be necessary to show that patient selection by PD-(L)1 molecular imaging results in equivalent or better patient outcomes than selection by IHC. Since molecular imaging offers non-invasive, real-time measurement of biomarkers, it may overcome the issues of dynamic changes in PD-L1 expression, which are often highlighted as the major challenge associated with this biomarker (1, 57, 97). Once this is established by prospective clinical trials, dissemination of PD-(L)1 imaging could likely be achieved relatively quickly because PET/CT imaging is technically mature and already in routine clinical use (67), minimizing the need for expensive equipment investment and extensive personnel training. Furthermore, in many countries there is already a well-established infrastructure for production and regional distribution of PET radiopharmaceuticals, such as ^18^F-FDG and ^68^Ga-DOTA-TATE (107, 108). This infrastructure could very likely also provide PD-(L)1 imaging agents to centers that provide PD-(L)1–targeted therapies. Global harmonization and approvals of imaging tests across regulatory bodies (109), as well as validation and standardization of PD-(L)1 imaging techniques, will be important for the technique is to gain widespread usage. Once implemented into clinical practice, molecular imaging is anticipated to improve patient care by minimizing ineffective therapy and over- or under-treatment. Early termination of clinical trials with drug candidates that have been identified as having poor safety or efficacy by molecular imaging is another area where these techniques can provide value.

Taking these challenges into consideration, there is a wealth of opportunity to expand the use of molecular imaging. Further advances are likely to take advantage of sequential PET tracer combinations; for example, to assess the expression of PD-L1 and of PD-1, as was carried out in the study conducted by Niemeijer et al. (72). Sequential PET tracer combinations have also been used to assess the correlation between metabolic activity and histopathology in glioma (110), and to assess myocardial viability and perfusion (111). Using combinations of tracers in such a way may allow a more comprehensive interrogation of selected (patho)physiology.

There may also be a role for PD-(L)1 PET tracers in characterizing changes in the TME in order to assess tumor progression, inflammatory responses, or drug resistance. For example, the radiation-associated abscopal effect can lead to T-cell infiltration of the TME by increasing the release of chemokines and expression of adhesion molecules, and upregulating class I major histocompatibility complexes, leading to immunologically cold tumors becoming immunologically hot tumors (112, 113). In this way, seemingly ICI-resistant tumors may begin to respond to such treatments. PD-(L)1 imaging could be used to visualize such events, allowing for a better understanding of the mechanisms of immuno-oncology and the principles underlying ICI/radiation combination therapies (113). Furthermore, by allowing the possibility to assess PD-(L)1 expression longitudinally and enabling the TME to be interrogated, molecular imaging is expected to facilitate the visualization of immunosuppressive cells, which may allow different types of progression, such as true progression and pseudoprogression, to be distinguished (57). However, given the complexity of the human immune system, a full understanding of the dynamic tumor microenvironment and the antitumor immune response will require comprehensive evaluation of other immune components in addition to PD-(L)1. Evaluation of cytokine signaling with a radiolabeled transforming growth factor (TGF)-β inhibitor, and SPECT imaging of tumor-infiltrating lymphocytes (with ^99m^Tc-labeled interleukin-2), regulatory T cells, and tumor-associated macrophages, are some of the developments beyond PD-(L)1 imaging that could contribute to improved assessment of response to ICI therapy and subsequent clinical management (42, 57).

It is likely that the role of molecular imaging for assessment of PD-(L)1 expression in a clinical setting will evolve alongside improved understanding of the PD-(L)1 pathway and other related immunobiology and biomarker technologies. Investigations have already pointed to the possible role of soluble PD-(L)1 detection in patient serum/plasma (114) and the use of AI-based digital pathology to assess PD-L1 expression (115, 116). The potential to multiplex these technologies will facilitate the acquisition of complex anatomic and pathologic patient data (34, 76). Molecular imaging may also be used in conjunction with other diagnostic methods, such as genomic and transcriptomic profiling, increasing the breadth of biological knowledge a clinician can obtain from a patient and aiding treatment strategies.

Molecular imaging, including PD-(L)1 PET imaging, will likely gain a more influential role in drug development in the future. Molecular imaging may be used in early-phase clinical trials to facilitate a more comprehensive understanding of the mechanisms of action of ICIs by enabling the assessment of receptor binding and biomarker accumulation (64, 65). Questions specific to a particular drug may be most effectively carried out by the drug developer, but both academic research groups and pharmaceutical companies could contribute to these studies. By understanding the uptake and distribution of drugs/molecules and their mechanisms of action, successful therapies may be identified earlier, with higher confidence (in “go”/”no-go” decisions), leading to lower development costs (64, 65).

In conclusion, PD-(L)1 molecular imaging offers the exciting opportunity to improve patient care by offering a non-invasive, dynamic technique to diagnose, select, and monitor patients based on PD-(L)1 expression, and to aid the development of immunotherapies.

## Author Contributions

All authors listed have made a substantial, direct, and intellectual contribution to the work, and approved it for publication.

## Conflict of Interest

DL, SB, and RS are employees of, and hold stock and shares in, Bristol Myers Squibb. WH was employed by Bristol Myers Squibb at the time the manuscript was drafted, and holds stock and shares in Bristol Myers Squibb. WW is on advisory boards for, and receives compensation from, Bayer, Blue Earth Diagnostics, Endocyte, ITM, and Pentixapharm, and has received research support from Bristol Myers Squibb, ImaginAb, Ipsen, and Piramal.

## Publisher’s Note

All claims expressed in this article are solely those of the authors and do not necessarily represent those of their affiliated organizations, or those of the publisher, the editors and the reviewers. Any product that may be evaluated in this article, or claim that may be made by its manufacturer, is not guaranteed or endorsed by the publisher.
