# Supplementary material for: Molecular Imaging and the PD-L1 Pathway: From Bench to Clinic
Source: Front Oncol. 2021 Aug 23;11:698425. doi: 10.3389/fonc.2021.698425 (PMC8420047; doi:10.3389/fonc.2021.698425)
Supplement: Supplementary file 1 [file Table_1.docx]

**Supplementary Table 1.** Examples of PD-(L)1 PET tracers tested in preclinical models.

| **Author, year** | **Tracer** | **Model(s)** | **Biodistribution/ accumulation** | **Key findings** |
| --- | --- | --- | --- | --- |
| **PD-L1 PET tracers tested in preclinical models** | | | | |
| Heskamp et al 2015 (1) | Anti-PD-L1.3.1 ^111^In | Breast cancer cell lines/mouse xenograft | Other organs did not show specific uptake of  ^111^In-PD-L1.3.1 | - ^111^In-PD-L1.3.1 bound specifically to PD-L1–expressing tumor cells and could discriminate between high and low PD-L1 expression - Radiolabeling did not reduce the immunoreactivity of ^111^In-PD-L1.3.1 - Clearance of the labeled antibody was slow - Correlation reported between tracer uptake and IHC, with discrepancies observed in some cell lines |
| Josefsson et al 2016 (2) | ^111^In-DTPA-anti-PD-L1 | Cell lines and mouse xenograft | Spleen, liver, thymus, heart, lungs, and in the spinal column | - Clearance of ^111^In-DTPA-anti-PD-L1 from all organs occurred within 144 hours - Radiolabeled anti–PD-L1 antibody yielded tolerable projected marrow doses, supporting its use for radiopharmaceutical therapy, although the marrow is likely to be the dose-limiting organ - Generally consistent with IHC. However, high uptake was observed in the thymus but was identified as having medium to low expression of PD-L1 by IHC |
| Chatterjee et al 2016 (3) | ^111^In-MPDL3280A  NIR-MPDL3280A | Cell lines and mouse (TNBC and NSCLC xenografts) | **TNBC xenograft:** lungs, liver, and spleen  **NSCLC xenograft:** liver and kidneys | - ^111^In-PD-L1-mAb and NIR-PD-L1-mAb were able to detect endogenous tumor PD-L1 expression - Xenograft uptake was validated with IHC for TNBC and NSCLC |
| Lesniak et al 2016 (4) | MPDL3280A ^64^Cu | Cell lines, orthotopic tumor models of human TNBC and in a syngeneic mouse mammary carcinoma model | **Xenografts:** liver, spleen, heart, lungs, kidneys, and thymus **Mammary carcinoma model (immunocompetent):** lymph nodes, heart, lungs, liver, and spleen | - ^64^Cu-Atezolizumab was able to detect varying levels of PD-L1 expression - Correlation was observed with IHC and flow cytometry (correlation coefficient *r* = 0.997) |
| Chatterjee et al 2017 (5) | ^64^Cu-WL12 | Cell lines (ovary and TNBC), mouse xenografts | Kidneys and liver | - Binding of ^64^Cu-WL12 to cancer cells *in vitro* was PD-L1 expression-dependent - Accumulation of ^64^Cu-WL12 was seen in tumors with high PD-L1 expression - ^64^Cu-WL12 binds PD-L1 rapidly and specifically - ^64^Cu-WL12 pharmacokinetics and biodistribution indicate that detection of PD-L1 is feasible within 60 minutes of radiotracer administration - IHC and the tracer provided consistent results |
| Nedrow et al 2017 (6) | ^111^ln-DTPA-anti-PD-L1 | Murine melanoma cell line, murine lymphoma cell line | Kidneys, liver, and spleen | - Thymus uptake of the labeled antibody was reported - The spleen was found to be a “sink” for the labeled antibody, affecting the amount of antibody in the TME - The thymus and brown adipose tissue had modest contrast to the background - Concordance with IHC was not determined |
| Trotter et al 2017 (7) | ^64^Cu-DOTA-22C3 ^18^F-AlF-NOTA-Z_PD-L1_1_ | Melanoma cell line, mouse xenograft | Kidneys and bladder | - High ^18^F-AlF-NOTA-Z_PD-L1_1_ uptake corresponded to areas staining positive in IHC using 22C3 - The fast blood clearance of ^18^F-AlF-NOTA-Z_PD-L1_1_ may have resulted in low target uptake but a high tumor-to-blood ratio - *Ex vivo* tracer signal was consistent with IHC carried out using the 22C3 antibody |
| Donnelly et al 2018 (8) | ADX_5322_A02 ^18^F (^18^F-BMS-986192) | Mouse (xenograft of lung and colon adenocarcinomas), cynomolgus monkey, human NSCLC tissue | Kidneys, urinary bladder, and spleen | - Increased accumulation of ^18^F-BMS-986192 was observed in xenografts expressing moderate levels of PD-L1 compared with control tumors with negligible PD‑L1 expression - Accumulation of ^18^F-BMS-986192 was observed in the spleen of a cynomolgus monkey - Concordance was observed between ^18^F-BMS-986192 and PD-L1 IHC staining in human NSCLC tissues. In cynomolgus monkeys, reduced accumulation in the spleen was consistent with PD-L1 staining assessed by IHC |
| Lv et al 2020 (9) | ^68^Ga-NOTA-Nb109 | A375 melanoma cell line (including human PD-L1 gene-transfected A375 cells), MCF-7 breast cancer cell lines, mice inoculated with A375 or MCF-7 | Relatively high uptake in the kidneys | - ^68^Ga-NOTA-Nb109 was found to clear quickly from the blood and have high *in vivo* stability - ^68^Ga-NOTA-Nb109 selectively accumulated in PD-L1–positive tumors - Tracer uptake correlated with PD-L1 expression determined by IHC |
| Stutvoet et al 2020 (10) | ^18^F‐BMS‐986192 | Human tumor cell lines, immunodeficient mice with human tumor cell line xenografts | Accumulation was seen in the kidneys, bladder, gall bladder, and in bone | - ^18^F‐BMS‐986192 could discriminate between various intrinsic levels of PD-L1 membrane expression - Binding by the tracer was specific to human PD-L1, with picomolar dissociation constants - ^18^F‐BMS‐986192 binding reflected levels of membrane, not cellular, PD-L1 expression - Imaging was conducted 60 minutes after tracer administration - Tracer binding was consistent with western blotting  and IHC |
| Li et al 2020 (11) | ^89^Zr-Avelumab | Breast cancer cell line MDA-MB-231,  MDA-MB-231 tumor-bearing mice | Uptake observed in the spleen and lymph nodes | - Uptake of ^89^Zr-Avelumab was reduced by blocking with unlabeled avelumab, demonstrating specific binding - Following blocking with unlabeled avelumab,  ^89^Zr-Avelumab had longer blood retention |
| **PD-1 PET tracers tested in preclinical models** | | | | |
| Maute et al 2015 (12) | HAC-PD-1 ^64^Cu | Colon cancer cell lines/mouse xenograft | Kidneys and liver | - HAC–PD-1 bound specifically to PD-L1–positive tumors and to areas that were inaccessible to antibody binding - A favorable signal-to-noise ratio was observed 1 hour post injection - HAC–PD-1 bound twice as many cells as a PD-L1 antibody - Concordance with IHC was not determined |
| Natarajan et al 2015 (13) | ^64^Cu-DOTA-PD-1 | Mouse splenic cells, CT26 cells, transgenic melanoma mouse | Lymphoid organs, liver, and kidneys | - Anti-PD-1 ^64^Cu exhibited high binding efficiency to the PD-1 receptor - Small animal PET/CT showed specific uptake of ^64^Cu‑DOTA-PD-1 in the tumor and lymphoid-associated organs - The tracer showed good tumor-to-background contrast - Western blotting and immunofluorescence showed concordance with tracer uptake in the spleen and tumor tissue |
| Cole et al 2017 (14) | ^89^Zr-Nivolumab | Cynomolgus monkeys | Liver, spleen, and lymph nodes (accumulation in lymph node over time) | - The accumulation of ^89^Zr-Nivolumab in the spleen was reduced using unlabeled nivolumab - Concordance with IHC was not determined |
| England et al 2017 (15) | ^89^Zr-Pembrolizumab | Two rodent models (mice and rats) and engrafted mice | **Mice:** liver and spleen tissues **Rats:** lungs, intestines, ovaries, and brain | - Specific binding of ^89^Zr-Pembrolizumab to activated T cells was observed in the humanized mouse model used in this study - Immunofluorescence was consistent with the PET tracer |
| Van der Veen et al 2020 (16) | ^89^Zr-Pembrolizumab | Humanized and non-humanized NOG mice xenografted with human A375M melanoma cells | High PD-1–mediated  uptake in immune  cell–containing tissues (including spleen, lymph nodes, and bone marrow) | - Uptake of ^89^Zr-Pembrolizumab in tumors was lower than in lymphoid tissues, but higher than other organs - Excess unlabeled pembrolizumab reduced uptake in lymphoid tissues but not in tumors |
| Li et al 2020 (17) | ^89^Zr-N-sucDf-Pembrolizumab | Healthy cynomolgus monkeys | High uptake in lymphoid tissues (including lymph nodes, spleen, and tonsils). Low uptake in the lungs, kidneys, muscle, brain,  and heart | - Co-administration of a large excess of unlabeled pembrolizumab reduced uptake in lymphoid tissues - ^89^Zr-N-sucDf-Pembrolizumab has the potential to be used to track the distribution of PD-1–expressing immune cells in humans and non-human primates |
| **PD-L1 and PD-1 PET tracers tested in preclinical models** | | | | |
| Mayer et al 2017 (18) | ^64^Cu-DOTA-HACA-PD1 ^64^Cu-NOTA-HACA-PD1 ^68^Ga-NOTA-HACA-PD1 ^68^Ga-DOTA-HACA-PD1 | Murine colon carcinoma cell line CT26, xenograft mouse model | **^64^Cu:** kidneys, bladder, lymph nodes, and salivary glands of the head, neck, and spleen **^68^Ga:** liver, spleen, and salivary glands | - Glycosylation affected tracer uptake, specificity, and clearance - PD-L1 expression in vitro was most accurately assessed by aglycosylated ^64^Cu-NOTA-HACA-PD1 - In *ex vivo* biodistribution studies, promising target-to-background ratios were observed for ^68^Ga-NOTA-HACA-PD1 and ^68^Ga-DOTA-HACA-PD1 - ^68^Ga-DOTA-HACA-PD1 demonstrated a high tumor signal and had low off-target accumulation - The HAC-PD1 radiotracer variants tested allowed detection of PD-L1 within 1 hour - Concordance with IHC was not determined |
| Hettich et al 2016 (19) | Anti-PD-1 ^64^Cu Anti-PD-L1 ^64^Cu | Mouse (melanoma xenografts) | Spleen, lymph nodes, and brown adipose tissue | - The tracers demonstrated high sensitivity and resolution - Concordance with IHC was not determined |

*ADX_5322_A02, anti–PD-L1 adnectin; AlF, aluminum fluoride; CT, computed tomography; DOTA, 1,4,7,10-tetraazacyclododecane-1,4,7,10-tetraacetic acid; DTPA, diethylenetriaminepentaacetic acid; FDG, fluorodeoxyglucose; HAC, high-affinity consensus; HACA, aglycosylated HAC; mAb, monoclonal antibody; IHC, immunohistochemistry; NIR, near infrared; NOG, NOD/Shi-scid/IL-2Rγ^null^; NOTA, 1,4,7-triazacyclononane-1,4-bis-acetic acid; NSCLC, non-small cell lung cancer; N-sucDf, N-succinyl desferal-Fe(III) ester; PD-1, programmed death-1; PD-L1, programmed death ligand 1; PET, positron emission tomography; TME, tumor microenvironment; TNBC, triple-negative breast cancer.*

**Supplementary Table 2.** Ongoing clinical trials assessing PD-L1 and PD-1 PET tracers for molecular imaging.

| **Tumor type** | **Trial identifier** | **Tracer used** | **Trial status** | **Number of patients enrolled** | **Other drugs/ compounds administered** | **Start date** | **Primary completion date** | **Study completion date** |
| --- | --- | --- | --- | --- | --- | --- | --- | --- |
| NSCLC | NCT03564197 | ^18^F-PD-L1 | Recruiting | 80 | Nivolumab or nivolumab/ chemotherapy combination | October 2018 | October 2020 | October 2020 |
|  | EUDRACT 2015-004760-11 | ^89^Zr-Nivolumab/ ^18^F-anti-PD-L1 | Ongoing | 10 | N/A | August 2016 | – | 2 years from last patient start |
|  | EUDRACT 2015-005765-23 | ^89^Zr-Durvalumab | Ongoing | 10 | N/A | – | – | 1 year from last patient start |
|  | NCT03065764/EUDRACT 2015-004260-10 | ^89^Zr-Pembrolizumab | Unknown^a^ | 10 | N/A | January 2017 | December 2019 | December 2019 |
|  | NCT03514719 | ^89^Zr-Avelumab | Recruiting | 37 | Avelumab | October 2018 | March 2022 | November 2022 |
|  | NCT02978196 | ^99m^Tc-NM-01 | Recruiting | 50 | N/A | February 2018 | June 2020 | August 2020 |
| Melanoma | NCT03520634 | ^18^F-BMS-986192 | Recruiting | 15 | N/A | May 2017 | October 2023 | October 2023 |
| Melanoma or NSCLC | NCT02760225 | ^89^Zr-Pembrolizumab | Active, not recruiting | 18 | N/A | October 2016 | March 2019 | December 2019 |
|  | NCT04193956 | – | Recruiting | 3,500 | Standard of care anti–PD-1 combination or monotherapy | August 2018 | July 2023 | July 2023 |
| Oral squamous cell carcinoma | EUDRACT 2018-002643-28/NCT03843515 | ^18^F-BMS-986192 | Recruiting | 15 | ^18^F-FDG, nivolumab | April 2019 | April 2021 | April 2022 |
| Breast cancer | EUDRACT 2019-001197-28 | ^89^Zr-Atezolizumab | Ongoing | 10 | Carboplatin | – | – | – |
| Diffuse large  B-cell lymphoma | NCT03850028 | ^89^Zr-Atezolizumab | Ongoing | 20 | Induction therapy  (R-CHOP), atezolizumab | May 2019 | April 2025 | April 2025 |
| Renal cell carcinoma | NCT04006522 | ^89^Zr-DFO-Atezolizumab | Active, not recruiting | 13 | N/A | October 2019 | July 2024 | July 2025 |
| Squamous cell carcinoma of the head and neck | NCT03829007 | ^89^Zr-Durvalumab | Recruiting | 58 | Durvalumab | April 2019 | November 2020 | March 2021 |
| Advanced PD-L1–positive malignancies, hepatocellular carcinoma, gastroesophageal junction adenocarcinoma,  gastric cancer | NCT03746704 | ^89^Zr-DFO-REGN3504 | Active, not recruiting | 28 | N/A | September 2019 | October 2022 | November 2022 |
| Advanced solid tumors | NCT02453984 | ^89^Zr-MPDL3280A-PET | Recruiting | 54 | MPDL3280A | February 2016 | September 2022 | September 2022 |
|  | NCT02478099 | ^89^Zr-MPDL3280A-PET | Recruiting | 98 | ^89^Zr-CD8,^18^F-FB-IL2, and MPDL3280A | February 2016 | September 2022 | September 2022 |
|  | NCT03638804 | ^89^Zr-KN035 | Recruiting | 10 | N/A | November 2018 | December 2019 | December 2019 |

Trials that are active or recruiting are shown. Data obtained from <https://clinicaltrials.gov/> and <https://www.clinicaltrialsregister.eu/ctr-search/search> using the search terms “imaging PD-L1”, “imaging anti-PD-L1”, “nuclear medicine anti-PD-L1”, “^89^Zr”, “^18^F”, “^99^mTc”, “^89^Zr PD-L1 tracer”, “^18^F PD-L1 tracer”, and “^99^mTc PD-L1 tracer”. Searches completed December 4, 2019.

^a^Completion date passed, status of trial not verified in >2 years.

*FDG, fluorodeoxyglucose; N/A, not applicable; NSCLC, non-small cell lung cancer; PD-1, programmed death-1; PD-L1, programmed death ligand 1; PET, positron emission tomography.*

# References

1. Heskamp S, Hobo W, Molkenboer-Kuenen JD, Olive D, Oyen WJ, Dolstra H, et al. Noninvasive imaging of tumor PD-L1 expression using radiolabeled anti-PD-L1 antibodies. *Cancer Res* (2015) 75(14):2928–36. doi: 10.1158/0008-5472.Can-14-3477

2. Josefsson A, Nedrow JR, Park S, Banerjee SR, Rittenbach A, Jammes F, et al. Imaging, biodistribution, and dosimetry of radionuclide-labeled PD-L1 antibody in an immunocompetent mouse model of breast cancer. *Cancer Res* (2016) 76(2):472–9. doi: 10.1158/0008-5472.Can-15-2141

3. Chatterjee S, Lesniak WG, Gabrielson M, Lisok A, Wharram B, Sysa-Shah P, et al. A humanized antibody for imaging immune checkpoint ligand PD-L1 expression in tumors. *Oncotarget* (2016) 7(9):10215–27. doi: 10.18632/oncotarget.7143

4. Lesniak WG, Chatterjee S, Gabrielson M, Lisok A, Wharram B, Pomper MG, et al. PD-L1 detection in tumors using [(64)Cu]Atezolizumab with PET. *Bioconjug Chem* (2016) 27(9):2103–10. doi: 10.1021/acs.bioconjchem.6b00348

5. Chatterjee S, Lesniak WG, Miller MS, Lisok A, Sikorska E, Wharram B, et al. Rapid PD-L1 detection in tumors with PET using a highly specific peptide. *Biochem Biophys Res Commun* (2017) 483(1):258–63. doi: 10.1016/j.bbrc.2016.12.156

6. Nedrow JR, Josefsson A, Park S, Ranka S, Roy S, Sgouros G. Imaging of programmed cell death ligand 1: impact of protein concentration on distribution of anti-PD-L1 SPECT agents in an immunocompetent murine model of melanoma. *J Nucl Med* (2017) 58(10):1560–6. doi: 10.2967/jnumed.117.193268

7. Trotter DEG, Meng X, McQuade P, Rubins D, Klimas M, Zeng Z, et al. In vivo imaging of the programmed death ligand 1 by (18)F PET. *J Nucl Med* (2017) 58(11):1852–7. doi: 10.2967/jnumed.117.191718

8. Donnelly DJ, Smith RA, Morin P, Lipovsek D, Gokemeijer J, Cohen D, et al. Synthesis and biologic evaluation of a novel (18)F-labeled adnectin as a PET radioligand for imaging PD-L1 expression. *J Nucl Med* (2018) 59(3):529–35. doi: 10.2967/jnumed.117.199596

9. Lv G, Sun X, Qiu L, Sun Y, Li K, Liu Q, et al. PET imaging of tumor PD-L1 expression with a highly specific nonblocking single-domain antibody. *J Nucl Med* (2020) 61(1):117–22. doi: 10.2967/jnumed.119.226712

10. Stutvoet TS, van der Veen EL, Kol A, Antunes IF, de Vries EFJ, Hospers GAP, et al. Molecular imaging of PD-L1 expression and dynamics with the adnectin-based PET tracer (18)F-BMS-986192. *J Nucl Med* (2020) 61(12):1839–44. doi: 10.2967/jnumed.119.241364

11. Li M, Ehlerding EB, Jiang D, Barnhart TE, Chen W, Cao T, et al. In vivo characterization of PD-L1 expression in breast cancer by immuno-PET with (89)Zr-labeled avelumab. *Am J Transl Res* (2020) 12(5):1862–72.

12. Maute RL, Gordon SR, Mayer AT, McCracken MN, Natarajan A, Ring NG, et al. Engineering high-affinity PD-1 variants for optimized immunotherapy and immuno-PET imaging. *Proc Natl Acad Sci U S A* (2015) 112(47):E6506–E14. doi: 10.1073/pnas.1519623112

13. Natarajan A, Mayer AT, Xu L, Reeves RE, Gano J, Gambhir SS. Novel radiotracer for ImmunoPET imaging of PD-1 checkpoint expression on tumor infiltrating lymphocytes. *Bioconjug Chem* (2015) 26(10):2062–9. doi: 10.1021/acs.bioconjchem.5b00318

14. Cole EL, Kim J, Donnelly DJ, Smith RA, Cohen D, Lafont V, et al. Radiosynthesis and preclinical PET evaluation of (89)Zr-nivolumab (BMS-936558) in healthy non-human primates. *Bioorg Med Chem* (2017) 25(20):5407–14. doi: 10.1016/j.bmc.2017.07.066

15. England CG, Ehlerding EB, Hernandez R, Rekoske BT, Graves SA, Sun H, et al. Preclinical pharmacokinetics and biodistribution studies of ^89^Zr-labeled pembrolizumab. *J Nucl Med* (2017) 58(1):162–8. doi: 10.2967/jnumed.116.177857

16. van der Veen EL, Giesen D, Pot-de Jong L, Jorritsma-Smit A, De Vries EGE, Lub-de Hooge MN. (89)Zr-pembrolizumab biodistribution is influenced by PD-1-mediated uptake in lymphoid organs. *J Immunother Cancer* (2020) 8(2). doi: 10.1136/jitc-2020-000938

17. Li W, Wang Y, Rubins D, Bennacef I, Holahan M, Haley H, et al. PET/CT imaging of 89Zr-N-sucDf-Pembrolizumab in healthy cynomolgus monkeys. *Mol Imaging Biol* (2020) 23(2):250–9. doi: 10.1007/s11307-020-01558-w

18. Mayer AT, Natarajan A, Gordon SR, Maute RL, McCracken MN, Ring AM, et al. Practical immuno-PET radiotracer design considerations for human immune checkpoint imaging. *J Nucl Med* (2017) 58(4):538–46. doi: 10.2967/jnumed.116.177659

19. Hettich M, Braun F, Bartholoma MD, Schirmbeck R, Niedermann G. High-resolution PET imaging with therapeutic antibody-based PD-1/PD-L1 checkpoint tracers. *Theranostics* (2016) 6(10):1629–40. doi: 10.7150/thno.15253
